# Using ChatGPT-Like Solutions to Bridge the Communication Gap Between Patients With Rheumatoid Arthritis and Health Care Professionals

**DOI:** 10.2196/48989

**Published:** 2024-02-27

**Authors:** Chih-Wei Chen, Paul Walter, James Cheng-Chung Wei

**Affiliations:** 1 National Applied Research Laboratories Taipei Taiwan; 2 National Council for Sustainable Development Taipei Taiwan; 3 Institute of Medicine Chung Shan Medical University Taichung Taiwan; 4 Faculty of Engineering Sciences University College London (UCL) London United Kingdom; 5 Faculty of Pharmacy Paris-Saclay University Orsay France; 6 Mines Saint-Etienne Saint-Etienne France; 7 Department of Allergy, Immunology & Rheumatology Chung Shan Medical University Hospital Taichung Taiwan

**Keywords:** rheumatoid arthritis, ChatGPT, artificial intelligence, communication gap, privacy, data management

## Abstract

The communication gap between patients and health care professionals has led to increased disputes and resource waste in the medical domain. The development of artificial intelligence and other technologies brings new possibilities to solve this problem. This viewpoint paper proposes a new relationship between patients and health care professionals—“shared decision-making”—allowing both sides to obtain a deeper understanding of the disease and reach a consensus during diagnosis and treatment. Then, this paper discusses the important impact of ChatGPT-like solutions in treating rheumatoid arthritis using methotrexate from clinical and patient perspectives. For clinical professionals, ChatGPT-like solutions could provide support in disease diagnosis, treatment, and clinical trials, but attention should be paid to privacy, confidentiality, and regulatory norms. For patients, ChatGPT-like solutions allow easy access to massive amounts of information; however, the information should be carefully managed to ensure safe and effective care. To ensure the effective application of ChatGPT-like solutions in improving the relationship between patients and health care professionals, it is essential to establish a comprehensive database and provide legal, ethical, and other support. Above all, ChatGPT-like solutions could benefit patients and health care professionals if they ensure evidence-based solutions and data protection and collaborate with regulatory authorities and regulatory evolution.

## Introduction

In recent years, the communication gap has led to intense relationships between patients and health care professionals. The use of ChatGPT-like solutions in health care has enormous potential to improve the patient-provider relationship, such as patient clinic letter writing [1], medical note-taking and consultation [2], and rheumatoid arthritis treatment. Although ChatGPT (OpenAI) [3] is not the only solution available, the technology has generated a lot of traction due to its advanced features, such as the ability to enhance rule-based chatbots.

However, it is important to note that ChatGPT-like solutions should not be viewed as a stand-alone solution but as an integrated interface in a larger ecosystem that allows access to multiple data sources. In terms of the patient-provider relationship, the use of ChatGPT can enable more fluid and effective communication between the 2 parties, which can improve the quality of care. In particular, the use of ChatGPT-like solutions in the context of methotrexate treatment for rheumatoid arthritis could have a significant impact. This viewpoint paper proposes a new relationship between patients and providers—“shared decision-making”; explains the potential of ChatGPT-like solutions in improving the patient–health care professional relationship from the clinical and patient perspectives; and suggests the importance of establishing a comprehensive database to promote the implementation of “shared decision-making” between patients and health care professionals.

## Toward Shared Decision-Making

In conventional medical settings, the relationship between patients and health care professionals was not equal, mainly because of the huge information gap between them, since patients lacked medical knowledge and decision-making capacity. In recent years, the rapid development of ChatGPT possesses enormous potential to bridge the information gap and improve the relationship between patients and health care professionals. For instance, ChatGPT could help provide the risk-benefit analysis of different treatment options, assisting health care professionals and patients to understand the advantages and disadvantages of each option and then make informed decisions together. It could also assist patients in understanding the complex medical jargon and technical details and provide information about the disease, treatment options, potential risks, and expected outcomes, allowing patients to participate in making informed decisions with health care professionals together. ChatGPT-like solutions allow bilateral communications between patients and health care professionals toward shared decision-making.

## Clinical Perspective

From the clinical standpoint, early diagnosis of rheumatoid arthritis is crucial [4] for health care professionals and should be based on clinical examinations and biological results such as serological tests [5]. However, the differential diagnosis of complex diseases such as rheumatoid arthritis–associated interstitial lung disease [6] remains a major concern [7], as it is responsible for a significant increase in mortality [8]. ChatGPT-like solutions could bring complementary support to diagnose the disease and predict its evolution. Thus, to the query “What could be the reason for cough and dyspnea in a patient with rheumatoid arthritis?” ChatGPT suggests interstitial lung disease in the first place. By integrating external data on risk factors (age, sex, and smoking), biological results (pulmonary function testing, autoantibodies, and biopsy), and imaging (high-resolution computed tomography and ultrasound) [6], ChatGPT-like solutions can assist in suggesting additional tests and confirming the diagnosis.

The initiation of treatment for rheumatoid arthritis should be in accordance with the latest official recommendations, such as those from the European League Against Rheumatism [9] and the American College of Rheumatology (ACR) [4]. An advanced tool such as ChatGPT provides clinicians with exhaustive information on the latest guidelines for the management of rheumatoid arthritis. For instance, if a clinician asks “What are the current guidelines for treating rheumatoid arthritis according to the ACR?” ChatGPT can retrieve the key points of rheumatoid arthritis management in accordance with the official ACR guidelines [4]. However, in the specific case of a request regarding recommendations for treating rheumatoid arthritis–associated interstitial lung disease, ChatGPT erroneously refers to nonexistent ACR guidance [10]. Currently, the tool has limitations, such as data exclusion after 2021 and response size limits.

The determination of a patient’s drug dose by the clinician is based on a comprehensive evaluation of the results of the biological tests and clinical examination. However, dose adjustment may not always be performed according to a standardized procedure and evidence-based solution, although this is crucial to ensure the effectiveness and tolerability of the treatment for the patient. Methotrexate is the most common treatment for rheumatoid arthritis, and an initial dose of 7.5-15 mg once a week is recommended, followed by a gradual increase in dose. However, poor patient adherence and nonpersistence to methotrexate therapy have been reported [11] mainly due to low dose tolerance. Optimization of methotrexate dose is therefore essential for treating rheumatoid arthritis [12]. The use of methotrexate monotherapy has shown similar efficacy to the combined use of methotrexate monotherapy with biologic disease–modifying antirheumatic drugs [13]. Process automation and integration of complementary data, based on solutions such as ChatGPT, could improve outcome prediction, contribute to drug dose optimization, and thus reduce costs to the health care system.

Access to information on ongoing clinical trials and their results would enable clinicians to propose treatments for people with rare conditions in rheumatoid arthritis. Compiling data on clinical trials and patient characteristics would allow clinicians to propose alternatives, for example, for patients who have failed current therapies. Identifying subpopulations would facilitate patient recruitment and bring more effective and safer drugs to market. However, one challenge is to deidentify data to comply with the US Health Insurance Portability and Accountability Act (HIPAA) [14]. As such, it is important for clinicians to prioritize patient privacy and confidentiality when accessing and using such data. In addition, it is necessary for further interdisciplinary research to improve the accuracy and persuasiveness of artificial intelligence (AI) chatbots to influence patients’ behaviors [15]. Moreover, the application of AI and machine learning in health care should still be regulated by establishing norms to reduce bias and reflect the real problems [16].

## Patient Perspective

From the patient’s perspective, it allows easy access to a large volume of information with a certain degree of scientific evidence, which improves the patient’s knowledge of rheumatoid arthritis and their health literacy. ChatGPT-like solutions thus contribute to dealing with the proliferation of unreliable sources of emerging information and widespread disinformation [17]. It is also a tool that could not only enable empowerment by acting interactively throughout the care pathway but also promote patient adherence to treatment. However, some concerns persist regarding the lack of supervision of this type of solution and the liability involved [18]. For example, in the case of methotrexate side effects, to the query “I have gastrointestinal problems and fatigue, is this related to my methotrexate intake?” ChatGPT suggests that the doctor can adjust the dosage. It does not provide suggestions to state that concomitant folate or folic acid changes would reduce toxicity. It also raises questions about the risk of patients adjusting their own dosage. ChatGPT-like solutions can strengthen expert patients’ collaboration, allowing the cocreation of care pathways; however, it can also be a source of conflict by pitting the tool’s and the caregiver’s advice against each other. Therefore, it is crucial to better supervise this tool from the beginning of its development, in order to clearly distinguish between its general public and medical use and to define the responsibilities of each. The use of ChatGPT-like solutions can improve communication and access to information for patients with rheumatoid arthritis but must be carefully managed to ensure safe and effective care.

A ChatGPT-like solution allows the patient to have continuous access to information in an interactive way that promotes understanding outside the clinical setting. This solution can play an important role in therapeutic education by providing information on the self-management of rheumatoid arthritis, on a drug such as methotrexate, or on the administration methods (oral and subcutaneous). Therefore, the query of “What precautions should be taken when taking methotrexate?” could instantly provide basic and exhaustive information (taking it with food, avoiding alcohol, staying hydrated, using contraceptives, etc) and could contribute to therapeutic education [19]. In addition, a ChatGPT-like solution could be used to communicate medical information on potential benefits and assist in administration [20], for example, when modifying the route of administration of methotrexate. This would have an impact on facilitating the acceptability of subcutaneous methotrexate, allowing better bioavailability and clinical efficacy. It would also reduce the time required to initiate treatment and avoid the use of biologics, thus having a significant impact on health care costs [21].

Further integration and analysis of patient requests would also accelerate the transition to more personalized medicine. ChatGPT-like solutions could identify patient profiles and adapt communication strategies to overcome resistance and nudge behavior. These solutions will have to be adapted to each country in terms of public health systems and beliefs.

## Establishment of a Comprehensive Database

The database is one of the critical elements of digital infrastructure for digital technology applications [22], especially AI-based solutions that require huge amounts of data to achieve more accurate results. However, using AI-based technology can be limited by the nontransparent learning process, difficulties in explanation and validation, and the influence of improper data [23]. Hence, the establishment of a comprehensive database, which is sourced from real-world data and updated on time and precisely, could contribute to overcoming limitations caused by insufficient data and support evidence-based clinical applications.

In recent decades, the Taiwan government launched the National Health Insurance (NHI) system that collected health-related data of health care providers, citizens, and legal residents. Since its establishment, the NHI database has been continuously improved by using the latest technologies to accommodate the increasing needs. During the COVID-19 pandemic, the NHI database successfully supported the Taiwan government in tracking patients, distributing face masks, and containing the infections [24,25].

On the other hand, using mobile health tools also contributes to the establishment of a comprehensive database. In recent years, tools such as the Apple Watch have been widely used to collect data about health conditions and identify possible illnesses of people. Mobile health tools allow the collection active data and passive data, which could better inform the health condition of the people [26].

Above all, the establishment of a comprehensive database is fundamental to applying AI-based solutions in the digital governance of health care. Moreover, applying AI-based solutions and other digital technologies should also be accompanied with comprehensive planning and flexible strategies to achieve effective digital governance in health care [22].

## Conclusions

In conclusion, ChatGPT-like solutions have the potential to improve the patient-provider relationship through “shared decision-making.” ChatGPT solutions should optimize the patient’s care pathway while improving the patient’s experience of using methotrexate in rheumatoid arthritis. However, there is a need to ensure evidence-based solutions and quantify these benefits. In the future, we may question the compatibility of the business model of mass-market solutions with health care system purposes, particularly concerning data protection. Using federated learning might be a way for developers to overcome this limitation. The implementation in a specific health care context should increase in the coming years with the development of solutions in specific domains such as Bio-Generative Pre-Trained Transformer. A deployment in clinical settings will require collaboration with regulatory authorities and potentially an evolution of the software as a medical device regulatory framework [27].

The need to include individuals in the design of these solutions is also crucial to consider from an efficiency point of view to avoid certain biases and from an ethical point of view. This solution also facilitates access to health care information for the entire world population in pursuit of the sustainable development goals set by the United Nations.

## Abbreviations

ACR: American College of Rheumatology
AI: artificial intelligence
HIPAA: Health Insurance Portability and Accountability Act
NHI: National Health Insurance
